# Vascular Endothelial Growth Factor Receptor Family in Ascidians, *Halocynthia roretzi* (Sea Squirt). Its High Expression in Circulatory System-Containing Tissues

**DOI:** 10.3390/ijms14034841

**Published:** 2013-03-01

**Authors:** Saeed Samarghandian, Masabumi Shibuya

**Affiliations:** 1 Division of Genetics, Institute of Medical Science, University of Tokyo, Tokyo 108-8639, Japan; E-Mail: samarghandians@mums.ac.ir; 2 Gakubunkan Institute of Physiology and Medicine, Jobu University, 270-1 Shinmachi, Takasaki, Gunma 370-1393, Japan

**Keywords:** vascular endothelial growth factor receptor, *Halocynthia roretzi*, sea squirt, circulatory system

## Abstract

The vascular endothelial growth factor (VEGF)-VEGF Receptor (VEGFR) system is an important pathway for regulation of angiogenesis. However, its evolutionary development, particularly the step from invertebrates to vertebrates, is still largely unknown. Here, we molecularly cloned the VEGFR-like gene from Halocynthia roretzi, a species belonging to the Tunicata, the chordate subphylum recently considered the sister group of vertebrates. The cDNA encoded a homolog of human VEGFR, including the transmembrane domain, and the tyrosine kinase domain with a kinase-insert region, which was designated S. sq VEGFR (GenBank AB374180). Similar to Tunicates including ascidians in the phylogenetic tree, the Amphioxus, another chordate, is located close to vertebrates. However, S. sq VEGFR has a higher homology than the Amphioxus VEGFR-like molecule (GenBank AB025557) to human VEGFR in the kinase domain-2 region. The *S. sq VEGFR* mRNA was expressed at highest levels in circulatory system-containing tissues, suggesting that S. sq VEGFR plays an important role in the formation or maintenance of circulatory system in Tunicates, *Halocynthia roretzi*.

Abbreviation

VEGF: Vascular endothelial growth factor
VEGFR: Vascular endothelial growth factor receptor
S. sq.: Sea squirt
*B. schlosseri*: *Botryllus schlosseri*
*H. roretzi*: *Halocynthia roretzi*
*C. intestinalis*: *Ciona intestinalis*

## 1. Introduction

**Figure 1:**
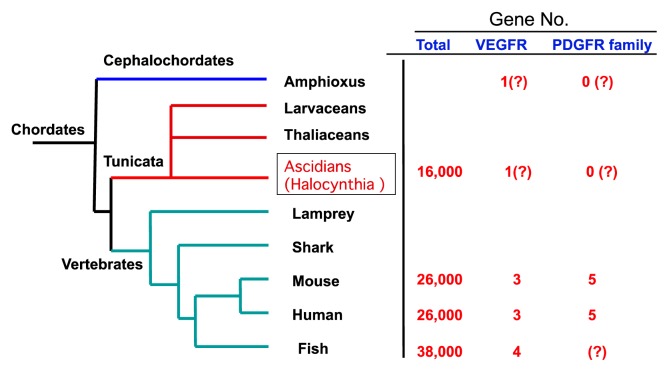
Phylogenic tree of the chordate and the number of genes for Vascular Endothelial Growth Factor receptor (VEGFR) and Platelet-derived growth factor receptor (PDGFR) family.

By comparing invertebrates’ genomes with those of humans and other animals, we could find new insights into the evolutionary origins of human tissues, as well as a better understanding of chordate and vertebrate development in general. The chordates have given rise to three groups: the vertebrates, *Cephalochordates* (*Amphioxus*), and *Tunicates*, which include the ascidians such as *Halocynthia roretzi* (*H. roretzi*) (Figure 1). The tunicates have recently emerged as a model system for understanding the organization of genomes. Because of their relative simplicity and position as an out-group to vertebrates, tunicates have a unique potential to illuminate the molecular mechanisms underlying the primitive body plan from which modern chordates diversified [1–6]. *H. roretzi* are bag-like gelatinous creatures that its body appears more primitive than creatures with backbones and spinal columns. Its genome is about 160 Mb in size, or one twentieth the size of the human genome, coding for about 16,000 genes [1,5]. The small size of the ascidian genome provides a distinct advantage for understanding genome organization and gene function [7,8]. Finding those genetic signals in the relatively simple animal could add to our knowledge of development such as heart and circulatory system in humans [9–14]. In Ascidians, several animals such as *Botryllus schlosseri* (*B. schlosseri*) and *Ciona intestinalis* (*C. intestinalis*) have been extensively studied on their development, morphogenesis and phylogeny. Particularly, *B. schlosseri* is unique in terms of their asexual growth of zooid and their circulatory system [10–12]. The circulatory system of *B. schlosseri* is divided into two: one is connected to heart and lacunae in various tissues in each zooid, and another consists of multiple ampullae and vessel system which is located at the peripheral area of a group of zooids.

Recent studies have identified several signaling pathways and regulatory factors governing angiogenesis in vertebrates. In 1990 we isolated a gene for novel receptor-type tyrosine kinase containing seven Immunoglobulin-like domains in the ligand-binding region from human tissue, and designated it as Fms-like tyrosine kinase-1 (Flt-1) (GenBank X51602) [15–17]. A few years later, de Vries *et al.* reported the binding of Flt-1 with Vascular Endothelial Growth Factor (VEGF), thus, Flt-1 is also known as VEGFR1 [18]. Two sequences distantly related to Flt-1/VEGFR1 were isolated from mammals; VEGFR2 (KDR/Flk-1) and VEGFR3 (Flt-4). We and others have demonstrated that these VEGFR family gene products have unique signaling pathways within the cell, and play crucial roles in angiogenesis and lymphangiogenesis [16,17,19]: Flt-1/VEGFR1 has very strong affinity to VEGF, but does a weak tyrosine kinase activity, showing dual (positive and negative) roles in angiogenesis. On the other hand, VEGFR2 has about one order-lower affinity to VEGF compared to Flt-1/VEGFR1, but has a 10-fold stronger tyrosine kinase activity, and transduces major angiogenic signals using Phospholipase Cγ-C-kinase-MAP kinase pathway for cell proliferation via the autophosphorylation of a single tyrosine (Y)-containing motif at the 1175Y site of carboxy terminal region [20,21].

However, understanding these interactions in details has been complicated by extensive gene multiplication events in vertebrates, and it is difficult to clarify their basal characteristics in circulatory system in the phylogenetic development [9–11,14,22–25]. To circumvent this genetic redundancy, we have focused on VEGFR in basal chordates. VEGFR/platelet-derived growth factor receptor (PDGFR)-related tyrosine kinase has been identified in Drosophila and other invertebrates including Cephalochordate (amphioxus) and Tunicates (ascidians) such as *B. schlosseri*, and *C. intestinalis*[12,26–29]. Therefore, we attempted to molecularly clone a receptor related to mammalian VEGFR from another popular ascidian, *H. roretzi*.

## 2. Results and Discussion

### 2.1. Isolation of VEGFR-Like Tyrosine Kinase Gene in *H. roretzi* by Using Degeneration-Primer Method

Total RNA from *H. roretzi* tissue was prepared by the AGPC method [30] (see Experimental Section). To test the quality of dsDNA, we selected Eph-Receptor as a positive control [31] well conserved among various species. By sequential PCR, we obtained a cDNA product of expected size and with the expected patterns of digestion by restriction enzymes.

**Figure 2:**
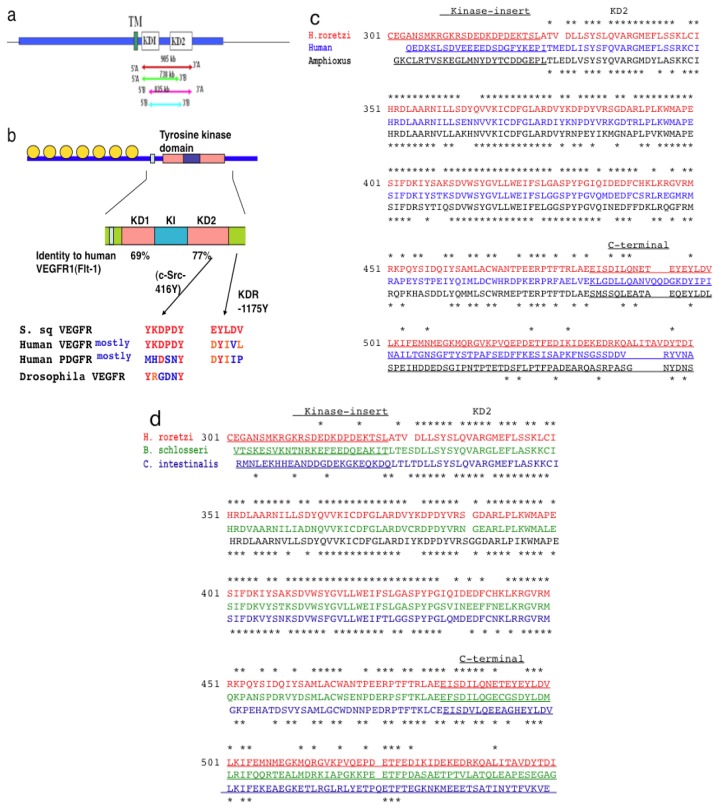
Amino acid sequence of S. sq VEGFR, and its strong homology with human VEGFR. (**a**) Isolation of cDNA related to *VEGFR* gene from ascidian, *H. roretzi*; (**b**) Schematic diagram of VEGFR of *H. roretzi* in comparison to human Flt-1 (VEGFR1). A high degree of conservation of critical tyrosine-containing residues in S. sq VEGFR and human VEGFR; (**c**) Sequence homology of S. sq VEGFR with human VEGFR1 in the KD2 region (157 amino acid-long sequence; ATVD—RLAE in S. sq VEGFR). [*]: identical amino acids with the VEGFR-related sequence in the middle; (**d**) Sequence homology among ascidian VEGFRs (S. sq VEGFR, *B. schlosseri* VEGFR, and *C. intestinalis* VEGFR) in the KD2 region. [*]: identical amino acids with the VEGFR-related sequence in the middle.

To test for the possible existence of VEGFR in *H. roretzi*, we made four degenerated oligonucleotide primers based on the sequence of the tyrosine kinase domain in *VEGFR* genes highly conserved among various species (Figure 2a). Once we obtained RT-PCR bands, we attempted a second PCR using another degenerated primer set at the inner sequences. We finally isolated a DNA sequence which encodes for tyrosine kinase with a kinase insert closely related to VEGFR (Figure 2b,c).

By using rapid amplification of cDNA and further extension of cDNA, we isolated a *H. roretzi* cDNA which corresponds to 557 amino acids (GenBank AB374180) (Figure 2b). The cDNA encodes the transmembrane domain, the entire tyrosine kinase domain with a 120-amino-acid-long kinase insert region, and the carboxyl tail (Figure 2b). Although we used a variety of degenerate primers, we could not find any other VEGFR-like sequences in *H. roretzi*. This strongly suggests that only one member of the VEGFR family exists in *H. roretzi*. Since “Sea squirt” is a popular name for ascidians, we designated the *H. roretzi* VEGFR-like sequence as S. sq VEGFR. The S. sq VEGFR showed a high homology with VEGFRs obtained from other ascidians, *B. schlosseri* and *C. intestinalis* (Figure 2d).

### 2.2. The Structure of *S. sq* VEGFR Is Closer to VEGFR Family than PDGFR Family in Humans

**Figure 3:**
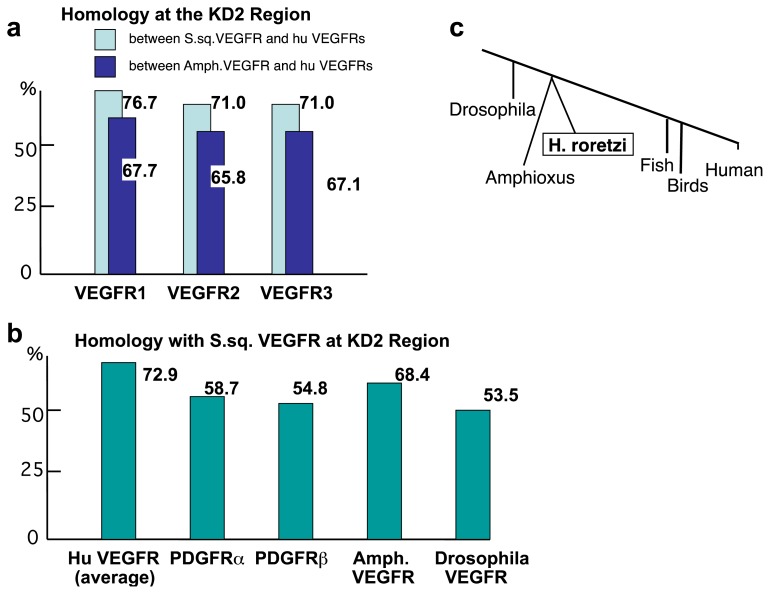
Human VEGFR is more homologous to S. sq VEGFR than to Amphioxus VEGFR-like protein. (**a**) Comparison of the KD2 domain among S. sq VEGFR, Amphioxus VEGFR-like molecule and human VEGFRs; (**b**) Comparison of KD2 domain in S. sq VEGFR with invertebrate VEGFRs and human VEGFRs as well as PDGFRs; (**c**) Diagram of Phylogenic tree (the order of ascidians such as *H. roretzi* and amphioxus is not completely fixed).

Although the *S. sq VEGFR* gene is not completely homologous with that of humans, the basic structure of tyrosine kinase relevant to its function is very well conserved. A sequence alignment of S. sq VEGFR *versus* human VEGFR1 [15] in different parts, *i.e.*, the kinase domain-1 (KD1), kinase-insert (KI) and kinase domain-2 (KD2), showed amino acid identity of 69%, 17%, and 77%, respectively (Figure 2c, data was not shown). As we found, the similarity was greatest at KD2. The sequence also shows homology with mammalian PDGFR, but less than with mammalian VEGFR (Figure 3). The higher homology of S. sq VEGFR with human VEGFRs than PDGFRs is consistent with the idea that this sequence encodes for a 7-Ig VEGFR-like molecule in ascidians, *H. roretzi* (Figure 2b).

Recently, the Amphioxus VEGFR-like sequence was published at the regions of a part of KI, KD2, and carboxyl tail (GenBank AB025557). The KD2 of human VEGFR1 shares 77%, 68% and 56%-identity with those of *H. roretzi*, Amphioxus, and Drosophila, respectively. Human VEGFR2 and VEGFR3 at the KD2 region also share more homology with S. sq VEGFR than with Amphioxus VEGFR (hVEGFR2 to S. sq VEGFR, 71% *vs.* hVEGFR2 to Amphioxus VEGFR, 65.8%; hVEGFR3 to S. sq VEGFR, 71% *vs.* hVEGFR3 to Amphioxus VEGFR, 67.1%) (Figure 3a). In the phylogenetic tree, based on the sequence homology of 18S rDNA, Amphioxus was previously suggested to locate closer than tunicates to vertebrates (Figure 3c) [32]. However, S. sq VEGFR has a higher homology in KD (Figure 3a,b) than the Amphioxus PDGFR/VEGFR-like molecule to human VEGFR.

### 2.3. Amino Acid Sequence Critical for the Intracellular Signaling of Human VEGFR Is Conserved in *S. sq* VEGFR

The “Y-K-D/N-P-D-Y” amino acid residue at KD2 of human VEGFR (residue 1048–1053 in human VEGFR1) is highly conserved among VEGFRs in vertebrates. VEGFR1 in mammals and birds have the sequence “Y-K-N-P-D-Y” whereas VEGFR2 and VEGFR3 in essentially all the vertebrates reported so far have “Y-K-D-P-D-Y”. The last “Y” corresponds to the 416-tyrosine in c-Src tyrosine kinase which is critical for activation of Src-kinase (Figure 2b). PDGFR family members have “M-R/H-D-S-N-Y” at this position, which is much diverged from the sequence in VEGFR. Interestingly, “Y-K-D-P-D-Y” is not well conserved in Drosophila VEGFR which has “Y-R-G-D-N-Y”. These results strongly suggest that the S. sq VEGFR has evolutionally developed to become closer to VEGFRs in vertebrates (Figure 2b).

We have recently shown 1175-Y in human VEGFR2, which corresponds to 1169-Y in VEGFR1, to be a site of autophosphorylation essential for the PhospholipaseCγ-PKC-MAP kinase pathway towards endothelial cell growth [20,21]. At the “D-Y-I-V-L” site in VEGFR2, S. sq VEGFR but not Drosophila VEGFR carries a similar sequence motif, “E-Y-L-D-V” (Figure 2b). This suggests that before vertebrates, S. sq VEGFR had already acquired a similar intracellular signaling system. The “Y-K-D-P-D-Y” motif in KD2, and “D-Y-I-V-L” motif in the 1175Y region are highly conserved in VEGFRs of other ascidians, *B. schlosseri* and *C. intestinalis*, with minor modifications (Figure 2d).

### 2.4. An Intimate Relationship between Expression of S. sq VEGFR mRNA and the Circulatory System in *H. roretzi*

**Figure 4:**
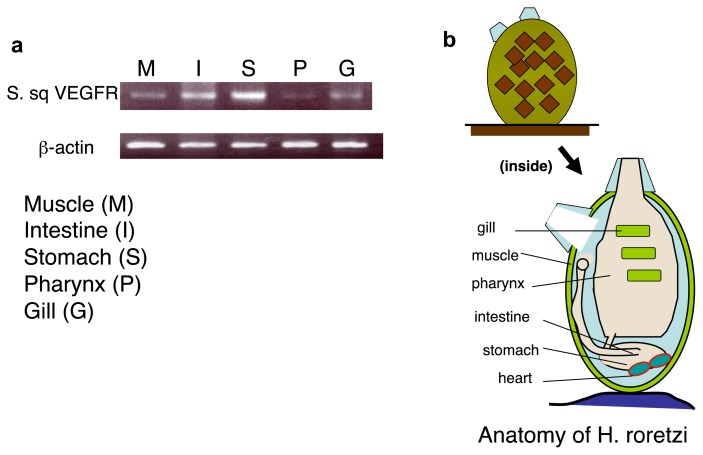
The expression of the *S. sq VEGFR* gene in the *H. roretzi* tissues is correlated with the existence of circulatory system. (**a**) RT-PCR analysis of *S. sq VEGFR* gene expression in the different *H. roretzi* tissues. Upper panel: PCR was performed using a *S. sq VEGFR*-specific primer set and reverse-transcribed cDNA from the total RNA of each tissue as the template. Lower panel: PCR was preformed using the *H. roretzi* β-actin-specific primer set and reverse-transcribed cDNA from total RNA of each tissue as the template (positive control); (**b**) Anatomical scheme of *H. roretzi*.

To see whether the levels of the *S. sq. VEGFR* transcript are tissue-specific or not in *H. roretzi*, we examined the expression patterns of *S. sq VEGFR* mRNA by using RT-PCR. The level of mRNA was highest in the stomach (heart-localized tissue), followed by the intestine and muscle, and lower in the pharynx and gill (Figure 4).

**Figure 5:**
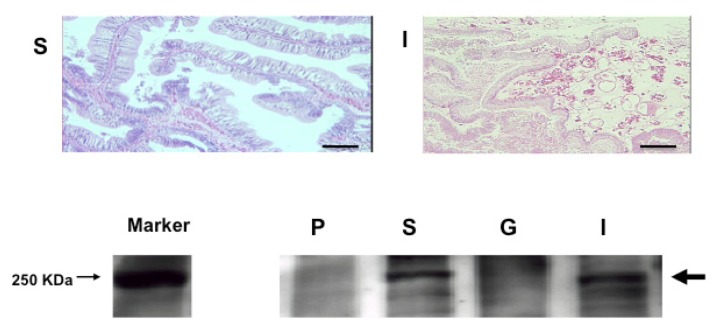
Strong expression of S. sq VEGFR protein in *H. roretzi* stomach (heart-localized tissue) and intestine. Upper part, Histology of the different tissues of *H. roretzi*. Lower part, Western blot analysis of S. sq VEGFR in different *H. roretzi* tissues. The tissues examined are: Intestine (I), Stomach (S), Pharynx (P) and Gill (G). Scale bar indicates 120 m.

A high level of expression of the *S. sq VEGFR* gene in the stomach (heart-localized tissue) suggests an intimate relationship between *S. sq VEGFR* mRNA and the circulatory system in this animal. Western blot analysis also revealed that the S. sq VEGFR is highly expressed in the stomach (including heart), and then intestine and significantly less abundant in gill, and pharynx (Figure 5). These results suggest that S. sq VEGFR plays a crucial role in the formation or maintenance of heart and vessel-like structure as well as blood cells in the latest stage of invertebrates.

The tunicate occupies a pivotal position in the animal kingdom for understanding the evolution of vertebrates. The ascidian genome provides an insight into the basic set of genes available at the very beginning of vertebrate evolution since the tunicates diverged just prior to the widespread gene duplication processes that are thought to have shaped and transformed the vertebrate genome [1–8,33].

Recently *VEGFR* mRNA of the *C. intestinalis* was reported (GenBank XM_002126535, in 2005). This sequence shows a high homology at amino acid levels in tyrosine kinase domain (KD1 and KD2), except for the kinase insert region, with that of the sequence S. sq VEGFR (*H. roretzi*) (Figure 2d). However, the *C. intestinalis* VEGFR-like sequence reported has very short, almost a half of, extracellular domain compared to those of human VEGFR and Drosophila VEGFR. This short sequence cannot contain seven Immunoglobulin-like domains which are important characteristics of VEGFR family. Thus, we suggest that this *C. intestinalis* VEGFR-like sequence is not for a typical VEGFR but a variant form.

More recently, Tiozzo *et al.* isolated a *B. schlosseri* cDNA encoding VEGFR-related molecule [12]. By using this sequence, they showed that the *VEGFR* mRNA is expressed in the epithelial cells covering the ampullae, and suppression of the gene expression via siRNA or human VEGFR-kinase inhibitor significantly attenuated the angiogenesis occurred after ampullaectomy and disorganized the whole morphology. These results suggest that VEGFR function is involved in a unique angiogenic process and morphogenesis in *B. schlosseri*.

In this study, we showed a higher homology of S. sq VEGFR (KD2 region) to human VEGFR compared to that of Amphioxus VEGFR to humans. This might indicate a strong phylogenetic affinity between tunicates and vertebrates. However, the information on the homology derived from a single gene product such as VEGFR is limited, and it could be modified with the structure of interacting molecule(s). Thus, the views on the relationships between the chordates should be carefully studied both at genome-wide level and each gene product level [13,14,34–37].

Both VEGFs and PDGFs and their receptors have been implicated in mammalian vascular development, and the prototype VEGF (VEGF-A) also regulates the development of hematopoietic cells. A VEGFR was found in Drosophila; however, there is no blood vessel structure in this animal. The VEGFR in Drosophila was shown to regulate the migration of border-cells, hemocytes, and thoracic epithelial cells [27,38,39].

To the authors’ knowledge, this is the first report for a VEGFR in Halocynthia roretzi that express significantly in the tissues containing hearts and circulatory system. However, it is not confirmed yet whether the S. sq VEGFR is expressed in the epithelial cells surrounding vessel-like structure and hematopoietic cells in this animal. Further analysis of this molecule in *Halocynthia roretzi* (S. sq VEGFR) should shed more light on the function of VEGFR in angiogenesis.

## 3. Experimental Section

### 3.1. Animals

*H. roretzi* was selected as the *ascidian* in our experiment. After the surface of each individual was washed with Ca^2+^ and Mg^2+^-free artificial sea water (460 mM NaCl, 9 mM KCl, 32 mM Na_2_SO_4_, 5 mM HEPES (pH 7.0), 5 mM EDTA-2Na, and 6 mM NaHCO_3_), the tunicate was removed and the exposed inner walls were opened. We mainly picked up heart (with part of stomach), intestine, gill, and pharynx for histological analysis.

### 3.2. The Acid Guanidium-Phenol-Chloroform (AGPC) Method

The AGPC method (Chomczynski and Sacchi 1987) [31] was used to isolate total RNA from different tissues of *H. roretzi*. Fresh tissue from different parts of *H. roretzi* was minced on ice and then homogenized at room temperature with 1 mL of denaturing solution (4 M guanidium thiocynate, 25 mM sodium citrate, pH 7; 0.5% sarcosyl, and 0.1 M 2-mercaptoethanol; solution D) in a glass-Teflon.

### 3.3. cDNA Synthesis

First-Strand cDNA was synthesized according to the protocol of the SMART™ cDNA Library Construction Kit (Clontech, CA, USA). Next, we amplified the cDNA by long-distance PCR (LD PCR). Then we purified the PCR product according to the protocol of the Qiaquick PCR Purification Kit (Qiagen, Hilden, Germany). Finally, cDNA size fractionation was carried out using CHROMA SPIN-400 columns according to the manufacturer’s protocol.

### 3.4. Degenerated Oligonucleotide Primers

#### (A) Primers for isolation of a VEGFR-like tyrosine kinase gene in *H. roretzi*

Degenerated oligonucleotide primers (5′A: 5′-TGGCCATGGLGCLTTYGG-3′; 5′B: 5′-ACAGTATGCMGCMGTLAASATG-3′; 3′A: 5′-GTCCAACATGSTYTMSTANA-3′, and 3′B: 5′-CAAAAAGCCARACSTCSCT-3′, wherein R is G or A; S is C or G; Y is C or T; L is A,C,G or T; M is A or C; N is C or G) were designed based on the sequences of *VEGFR* genes highly conserved among various species.

#### (B) Primers for detection of β*-actin* mRNA in *H. roretzi*

Degenerated oligonucleotide primers (5′A: 5′-TGAAGCCAGAGCAAGAGAGG-3′; 5′B: 5′-GTATCGTAACCAACTGGGACG-3′; 3′A: 5′-TTTGCTGATCCACATCTGTTG-3′; 3′B: 5′-TTAGAAGCATTTGCGGTGAAC-3′) were designed based on the sequence of β*-actin* genes highly conserved among various species. Rapid amplification of cDNA ends of the 5′-or 3′-end was performed with these degenerate primers and anchor primers corresponding to the anchor sequence combined with the 3′-end of the oligo(dT) primer or the sequence involved in the 5′-end of the SMART IV oligonucleotide. The nucleotide sequences were determined using a BigDye Terminator cycle sequencing kit and an ABI310 automated DNA sequencer (Applied Biosystems, Foster City, CA, USA). To further extend the S. sq VEGFR sequence, we generated several primers based on partial sequences of S. sq VEGFR determined as the original, in both the 5′ and 3′ direction.

### 3.5. PCR

PCR was performed in a 20 μL reaction mixture containing 1 μL of cDNA, 5 μL of 10+PCR buffer, 0.2 mM of each dNTP, 1 mM of MgSO_4_, and 15 pmol of each primer. PCR was performed with one cycle of incubation at 94 °C for 2 min, followed by 35 cycles at 94 °C for 15 s and 50 °C for 30 s, with a final cycle at 68 °C for 5 min. The PCR products were separated by electrophoresis in a 1.5% agarose gel and then purified by gel extraction. Sequencing was performed with a PE-Biosystems BigDye terminator kit and an ABI Prism 377 XL sequenator.

### 3.6. Restriction Enzymes

HaeIII, Saw3AI, HinfI, and SspI as restriction enzymes have been used for checking the existence of Eph receptors as a positive control in *H. roretzi*.

### 3.7. Histological Analysis

Tissue samples were fixed in 4% paraformaldehyde (PFA). For Hematoxylin and Eosin staining, paraffin-embedded specimens were sectioned at a thickness of 5 μm and stained with hematoxylin and eosin using a standard protocol.

### 3.8. RT-PCR Detection of S. sq VEGFR mRNA

Total RNA samples were exacted from the different *H. roretzi* tissues (Heart with stomach tissue, intestine, pharynx, gill and muscle) using the AGPC method [31]. Reverse transcription was performed using the SuperScript II reverse transcriptase (Invitrogen, Carlsbad, CA, USA) and dT 15 primer. PCR assay was carried out to detect the expression of the S. sq VEGFR gene in tissues. The PCR mixture contained reverse-transcribed DNA from the total RNA samples of ascidian tissues, Fw primer (50 pmol), Rw primer (50 pmol), dNTPs (20 pmol), PCR buffer, and rTaq DNA polymerase (TaKaRa) in 100 μL. The PCR conditions used were as follows: 94 °C for 4 min; 30 cycles at 94 °C for 30 s, 50 °C for 30 s, and 72 °C for 30 s; and a final extension at 72 °C for 5 min. PCR with the β-actin Fw primer (5′TGAAGCCCAGAGCAAGAGAGG-3′) and β-actin Rw primer (5′CGTTCTTGATTCTGGCGATGG-3′) was used to detect the expression of the β*-actin* gene in the ascidian tissues, as the positive control. After finding the sequence of β-actin in *H. roretzi*, we generated a pair of primers from the β-*actin* sequence for RT-PCR (Fw, 5′TGAAGCCCAGAGCAAGAGAGG-3′, Rw, 5′CGTTCTTGATTCTGGCGATGG-3′).

### 3.9. Generation of Antibody

To generate antibody against the S. sq VEGFR protein, the 20-amino-acid peptide “CEGANSMKRGKRSDEDKDPD” which corresponds to a part of the kinase-insert region within the tyrosine kinase domain, was synthesized and conjugated with the KLH carrier. Rabbits were immunized several times with the conjugated protein, and antiserum was obtained.

### 3.10. Western Blotting

Proteins separated by SDS-PAGE were transferred to Immobilon P membrane. The membrane was blocked with 5% BSA in PBST buffer and incubated with purified primary antibody for 2 h. Corresponding horseradish peroxidase-conjugated secondary antibodies were used for detection of the primary antibody with chemiluminescent reagent.

DNA Data Base Accession number for S. sq VEGFR: GenBank AB374180.

## 4. Conclusions

For the first time, we molecularly cloned the VEGFR-like gene in *Halocynthia roretzi*, one of three basal chordates. S. sq VEGFR has a higher homology than the Amphioxus VEGFR-like molecule to human VEGFR. The *S. sq VEGFR* mRNA was expressed at higher levels in heart-localized tissue (stomach) and circulatory system-containing tissue (intestine), suggesting that S. sq VEGFR plays an important role in the formation or maintenance of circulatory system in tunicates, *H. roretzi*.
